# Gender differences in the association between sleep duration and diabetes in Chinese adults

**DOI:** 10.3389/fendo.2025.1385618

**Published:** 2025-05-20

**Authors:** Haiyan Yu, Xiaodong Zhang, Haishan Wei, Yuan Guo, Hao Wu

**Affiliations:** ^1^ School of General Practice and Continuing Education, Capital Medical University, Beijing, China; ^2^ Department of General Practice, Qilu Hospital of Shandong University, Jinan, Shandong, China; ^3^ Department of General Practice, Zhongshan Hospital, Fudan University, Shanghai, China

**Keywords:** sleep duration, new-onset diabetes, gender differences, non-linearity, Chinese adults

## Abstract

**Background and objectives:**

There is growing evidence that sleep duration is associated with future risk of new-onset diabetes mellitus. However, discussions of gender differences have yielded inconsistent results. The aim of this longitudinal study was to explore this issue in a large group of Chinese adult population.

**Methods:**

13,142 participants (6,366 men and 6,776 women) without diabetes at baseline from China Health and Nutrition Survey (CHNS) were included. They participated in at least two rounds of the CHNS during 2004-2015. Multivariate Cox proportional hazards regression models were used to calculate hazard ratios (HRs) and 95% confidence intervals (CIs) for new-onset diabetes. In addition, non-linearity relation of sleep duration and new-onset diabetes was analyzed by restricted cubic splines (RCS).

**Results:**

During the follow-up period from 2004 to 2015, there were 222 new cases of diabetes in men (5.34 per 1000 person-years) and 234 cases in women (5.26 per 1000 person-years) participants had newly developed diabetes. In women, there was a U-shaped association between sleep duration and new-onset diabetes with the lowest risk for diabetes in individuals sleeping 8-9h per day after adjusting for covariates. Compared with the reference (8-9h/day), the HRs for participants who slept <6h/day, >10h/day were 2.47 (1.22-4.99), 2.65(1.14-6.16) after adjustment for covariates. Among men <60 years old, compared with subjects who slept <7 hours per day, those slept 7-<8h/day (HR = 0.73, 95%CI 0.42-1.24), 8-<9h/day (HR = 0.57, 95%CI 0.33-0.99), and ≥9 h/day (HR = 0.35, 95% CI 0.14-0.90) had lower risk of diabetes after adjusting for all potential confounders (*p*-trend < 0.001). No significant relationship between sleep duration and diabetes was observed in men over 60 years of age.

**Conclusions:**

There was a U-shaped association between sleep duration and the risk of diabetes in women, with the lowest risk for diabetes at approximately 8–9h/day. For men, risk for new-onset diabetes decreased significantly with increasing sleep duration only among participants < 60 years. The effect of sleep on older men was not statistically significant.

## Highlights

- The large Chinese population investigation and follow-up of sex differences in the associations between sleep duration and incidence of diabetes.
- There was a U-shaped association between sleep duration and the risk of diabetes in women.
- Risk for new-onset diabetes decreased significantly with increasing sleep duration only among men < 60 years.
- Gender-specific diabetes prevention strategies are offered.

## 1 Introduction

Diabetes mellitus is a chronic metabolic disease. Diabetes and its numerous complications are severe global public health burden (1). In 2017, there were approximately 425 million adults with diabetes worldwide, and this is expected to rise to 642 million by 2040. Currently, about 1 in 11 adults worldwide suffer from type 2 diabetes (2). China has the largest diabetes population in the world and the prevalence is on the rise. Diabetes is severe public health burden in China (3, 4). Therefore, exploration of more modifiable risk factors is crucial for the primary prevention of diabetes. Traditional diabetes prevention and management focuses on controlling weight, increasing physical exercise, adopting proper dietary habits and using medication regularly. In recent years, the relationship between sleep and diabetes has been increasingly studied (5–7).

Sleep is an essential part of everyone’s life, occupying a third or more of the day. Sleep duration is strongly associated with new-onset diabetes and other diseases. Some meta-analyses have reported a U-shaped association between sleep duration and diabetes, implying that both short and long sleep duration are risk factors for diabetes (5, 8). What’s more, a cohort study with a mean follow-up of 7.9 years showed that long sleep duration was associated with a higher risk of diabetes, but not with short sleep duration (9). However, another new study found that accelerometer-measured short but not long sleep duration was associated with a higher risk of incident type 2 diabetes (7). Such inconsistency in the results of the studies above may be due to gender differences. A study have found that compared with women, men may show more sleep-related problems, such as sleep disapnea, easy waking at night, obstructive sleep apnea-hypopnea syndrome, etc (10). Another study has found the opposite result, that compared with men, women have more significant sleep problems, such as lower sleep efficiency, higher daytime sleepiness, and worse sleep quality (11). There was also study that found an independent association between sleep duration and type 2 diabetes in middle-aged women, while the trend in men was not statistically significant (12). One study showed that only women were sensitive to short sleep duration while men were not (13). However, that study did not further clarify the relationship between sleep and new-onset diabetes in men. Moreover, some studies have suggested that recommendations for appropriate sleep duration should be sex-specific (14, 15).

Therefore, in order to provide scientific evidence for research on this issue, we utilised data from Chinese adults in the 2004-2015 China Health and Nutrition Survey (CHNS) to conduct a large national cohort study.

## 2 Methods

### 2.1 Participants

China Health and Nutrition Survey (CHNS) database is a constant, open of, longitudinal queue survey, 1989 years so far has completed the ten-round survey (1989 1991 1993 1997 2000 2004 2006 2009 2011 and 2015). The sampling process consisted of five stages. In the first stage, 15 provinces in China were randomly selected. In the second stage, a multistage random clustering method was used to select samples from each province. In the third stage, stratified by income (low, medium and high), 4 counties and 2 cities were randomly selected from each province by using a weighted sampling scheme. In the fourth stage, rural communities in the county and suburban communities in the city were randomly selected. Twenty households were randomly selected in each community in the final stage and all family members were interviewed. The present study is based on 5 rounds of CHNS data from 2004 to 2015. Participants with the following were excluded: participants who were pregnant, < 18 years of age, with missing diabetes diagnosis, with incomplete records of sleep duration, with only one survey wave, with diabetes at baseline, with cancer or cardiovascular diseases or stroke at baseline and with extreme dietary energy data (male: > 4200 kcal or < 600 kcal, female > 3600 kcal or < 500 kcal). Finally, a total of 13142 participants in the formal analysis was enrolled (**Supplementary Figure 1**).

This survey was approved by the institutional review boards of the University of North Carolina at Chapel Hill and the National Institute of Nutrition and Food Safety, and the Chinese Center for Disease Control and Prevention. Each participant voluntarily signed a written informed consent before recruitment. All methods were performed in accordance with the relevant guidelines and regulations. The data and study materials that support the findings of this study can be found on the official website of CHNS (http://www.cpc.unc.edu/projects/china).

### 2.2 Measurement of sleep duration

Sleep duration was derived from a self-report questionnaire that included the question “How many hours each day do you usually sleep, including during both daytime and nighttime?”.

In this study, the cumulative average length of sleep for each participant during the last round visit period from baseline to the date of new-onset of diabetes or the end of follow-up was calculated to represent long-term sleep duration and minimize inter-individual differences.

### 2.3 Additional variables

All covariate information was obtained from questionnaires, including age, education level, residence, smoking, alcohol consumption, tea and coffee consumption, dietary intake. Due to the relatively large number of missing data values, we included physical activity and socioeconomic status into the sensitivity analysis after multiple interpolation. Age was divided into two groups according to <60 years old and ≥60 years old. The residential areas are divided into urban and rural areas. The level of education is divided into illiteracy, elementary school, middle school and high school and above. Participants who had consumed any alcoholic beverage more than once a month in the past were classified as drinkers. Participants who had smoked cigarettes in the past or were currently smoking were classified as smokers. Smoking, alcohol consumption, tea and coffee consumption were all classified as yes or no. Dietary intake is the average of an individual’s dietary intake, including protein intake, fat intake, energy intake, and carbohydrate intake, collected over three consecutive days. Physical activity level is defined as the time spent on self-reported physical activity multiplied by the product of a specific metabolic equivalent value, expressed as metabolic in counts of tasks (MET) -hours/week, divided into low, medium, and high groups (16). We defined socioeconomic status based on occupational titles, which were divided into three groups: low (e.g., farmer, general worker, etc.), medium (e.g., clerk, salesman, foreman, etc.), and high (e.g., professor, officer, director, etc.) (17). Body mass index (BMI) was calculated as weight (kg) divided by height squared (m^2^). For all covariates, we used the baseline year measurements. After the participants rested for five minutes, blood pressure was measured in a standardized manner by trained researchers using a mercury manometer. Mean systolic and mean diastolic blood pressures from three independent measurements in the same arm were used.

### 2.4 Outcome

The study outcome was defined as new-onset diabetes diagnosed by an experienced physician during the follow-up period and did not differentiate between types. Through the interview in the questionnaire survey: “has a doctor ever told you that you suffer from diabetes?” Participants who answered “yes” were defined as having new-onset diabetes. If no diabetes was found in all subsequent surveys, the follow-up time was calculated based on the last survey date.

### 2.5 Statistical analysis

All continuous variables were expressed as mean (SD) or medians (interquartile ranges), and categorical variables were expressed as number (n) and percentage (%). Wilcoxon rank-sum test or ANOVA test for continuous variables and Chi-square test for categorical variables were used to compare the difference between the non-diabetes and diabetes groups.

The year in which participants were first enrolled in the survey was considered baseline. The follow-up year for each participant was calculated from baseline to the first diagnosis of diabetes (the intermediate date between the survey of the first diagnosis and the previous most recent survey), the last round of surveys before participants left the survey or the end of the latest survey (2015), whichever came first. Incidence rates of diabetes, expressed as per 1000 person-years, were calculated as the number of new-onset diabetes cases divided by the person-years of follow-up.

The restricted cubic spline is a commonly used method for fitting nonlinear relationships, which is used to examine nonlinearity and explore the shape of the dose-response relationship between sleep time and new-onset diabetes risk. After adjusting for age, education, urban and rural areas, BMI, systolic, smoking, alcohol, tea, coffee, energy intake, fat intake, carbohydrate intake and protein intake, the association between sleep duration and new-onset diabetes was analyzed using Cox proportional hazards models. In addition, Kaplan-Meier curves were performed to describe the cumulative incidence of diabetes and differences between groups were estimated with the log-rank test. A two-tailed P <0.05 was considered to be statistically significant in all analyses. All statistical analyses were performed with STATA (version 17.0) and R software (version 4.2.3).

## 3 Results

### 3.1 Basic characteristics of study participants

The mean age of the 13,142 enrolled patients was 46.5 ± 14.8. The incidence rate of diabetes was 5.34/1000 person-years for men and 5.26/1000 person-years for women.

**Table 1** shows the baseline characteristics of the participants. Participants who eventually developed diabetes were older, tended to have higher BMI, SBP, DBP, lower educational levels and sleep duration, and lived in urban area. In addition, the male participants with incidence of diabetes were had higher rates of tea consumption. They had higher fat intake and lower carbohydrate intake in dietary intake. In women, participants who showed progression to diabetes had lower carbohydrate intake.

**Table 1:** Baseline characteristics of participants.

Characteristics	Male (6366)			Female (6776)		
	Non-diabetic	Diabetic	p	Non-diabetic	Diabetic	p
N	6366	222		6776	234	
Age, year	46.1 (15.0)	53.3 (11.0)	<0.01	46.2 (14.7)	57.7 (10.3)	<0.01
Education Level (%)			0.87			<0.01
Illiteracy	570 (9.3)	21 (9.5)		1495 (22.9)	77 (32.9)	
Primary school	1228 (20.0)	40 (18.0)		1365 (20.9)	56 (23.9)	
Middle school	2245 (36.5)	86 (38.7)		1938 (29.6)	57 (24.4)	
High school or above	2101 (34.2)	75 (33.8)		1744 (26.7)	44 (18.8)	
Urban (%)	2299 (37.4)	105 (47.3)	<0.01	2481 (37.9)	121 (51.7)	<0.01
BMI, (kg/m2)	23.2 (3.3)	25.6 (3.2)	<0.01	23.2 (3.4)	26.4 (3.9)	<0.01
SBP, mmHg	123.8 (16.3)	131.9 (17.6)	<0.01	119.7 (18.7)	134.7 (19.5)	<0.01
DBP, mmHg	80.2 (10.5)	84.0 (10.5)	<0.01	77.0 (10.9)	84.3 (12.2)	<0.01
Smoking (%)	3868 (63.1)	134 (60.4)	0.41	241 (3.7)	14 (6.0)	0.07
Drinking alcohol (%)	3623 (59.8)	116 (53.2)	0.05	630 (9.7)	19 (8.2)	0.44
Drinking tea (%)	3113 (50.7)	131 (59.0)	0.02	1903 (29.1)	73 (31.2)	0.49
Drinking coffee (%)	237 (3.9)	6 (2.7)	0.38	280 (4.3)	7 (3.0)	0.33
Dietary intake						
Energy, Kcal/day	2226.3 (1791.0,2695.6)	2238.2 (1713.5,2779.6)	0.98	1890.4 (1507.0,2288.1)	1811.8 (1462.8,2152.2)	0.07
Carbohydrate, g/day	309.3 (236.7, 389.8)	297.4 (221.0, 371.2)	0.04	261.7 (201.4, 332.3)	239.0 (189.6, 313.7)	0.01
Fat, g/day	69.5(46.7,95.4)	75.1(52.7,101.4)	0.01	60.8(40.6,83.8)	61.4(42.5,86.4)	0.40
Protein, g/day	68.5(53.9,84.3)	70.9(54.8,89.4)	0.23	58.5(46.1,72.2)	58.5(45.0,73.5)	0.96
Sleep duration, hours/day	7.9 (0.9)	7.8 (0.8)	0.01	7.9 (0.9)	7.7 (1.1)	<0.01

### 3.2 Correlation between sleep duration and new-onset diabetes in female group

**Figure 1:**
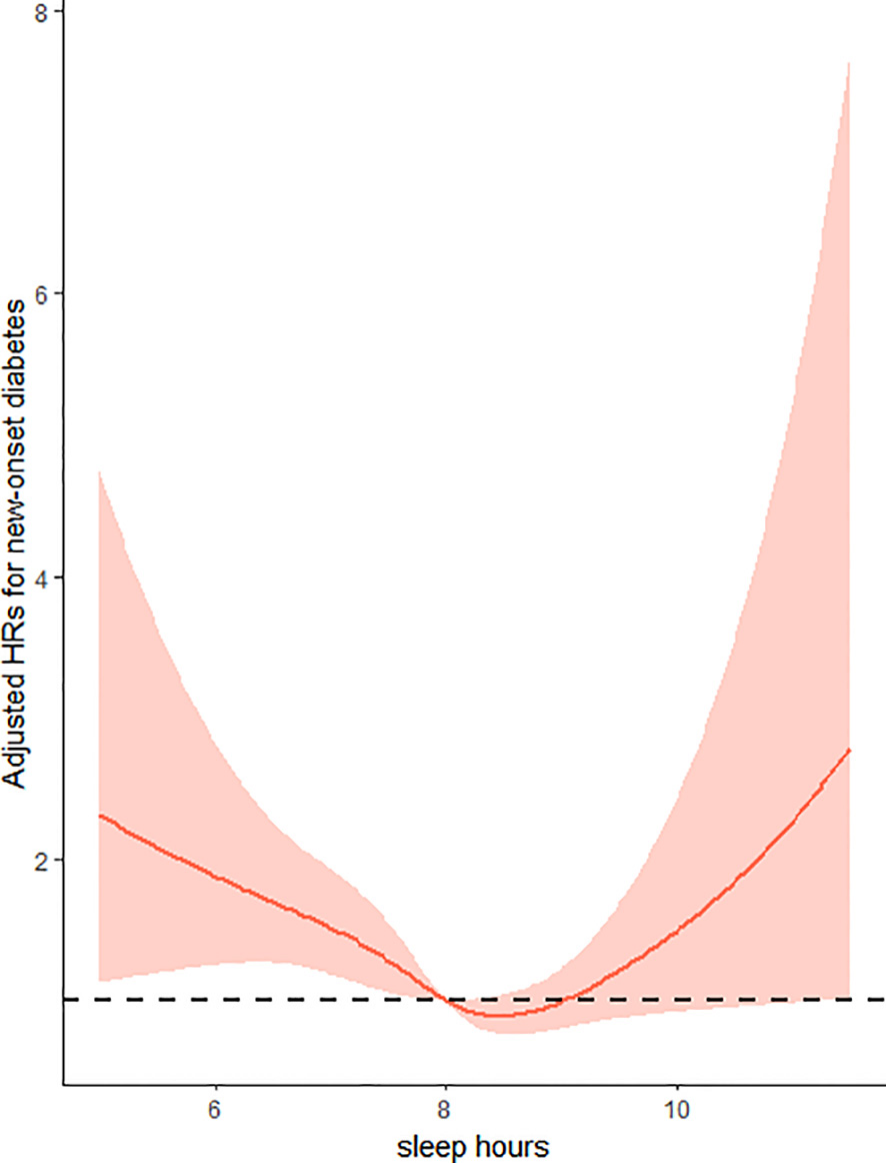
Restricted cubic splines of sleep hours associated with risk of new-onset diabetes in women. Adjusted for age at baseline (<60 years, ≥60 years), residence (urban or rural) and education (illiteracy, primary school, middle school, high school or above), BMI (<24kg/m2,≥24kg/m2), SBP (<140mmHg,≥140mmHg), smoking status (yes/no), alcohol consumption(yes/no), drinking tea(yes/no), drinking coffee(yes/no), total energy intake (continuous), total fat intake (continuous), total carbohydrate intake (continuous) and total protein intake (continuous).

Overall, there was a U-shaped relationship between sleep duration and new-onset diabetes in female group, with a nadir at approximate 8-9h/day (**Figure 1**, p for non-linearity =0.0072). As sleep duration increased, the risk of new-onset diabetes decreased significantly in women with ≤ 8 hours of sleep per day and increased in participants with ≥ 9 h/day of sleep.

In addition, we also included the sleep duration as a categorical variable divided into six groups in the regression models. Consistently, compared to people who slept for 8-9 h/day, the hazard ratios (HRs) and 95% confidence intervals (CIs) of new-onset diabetes were 2.47(1.22-4.99)for <6 h/day, 1.78(1.17-2.72)for 6-<7h/day, 1.33(0.98-1.81)for 7-<8h/day, 0.71(0.33-1.56)for 9<-10h/day, and 2.65(1.14-6.16)for>10h/day, respectively, after adjusting for age, region, education level, BMI, SBP, smoking status, drinking status, drinking tea, drinking coffee, and dietary intake (Energy, Carbohydrate, Fat, Protein) (**Table 2**).

**Table 2:** Relationship between sleep duration (categorical variable) and new-onset diabetes in women.

Sleep duration	Incidence rate/1000 person-years (Person-years)	Model1		Model2		Model3	
		HR (95% CI)	P	HR (95% CI)	P	HR (95% CI)	P
<6h	19.34 (454.5)	4.70 (2.36-9.33)	<0.001	2.78 (1.39-5.58)	<0.001	2.47 (1.22-4.99)	0.012
6h-<7h	12.74 (2591)	3.14 (2.11-4.69)	<0.001	2.19 (1.45-3.30)	0.004	1.78 (1.17-2.72)	0.008
7h-<8h	5.77 (15586)	1.47 (1.10-1.98)	0.01	1.36 (1.01-1.83)	0.042	1.33 (0.98-1.81)	0.062
8h-9h	3.94 (22357.5)	Ref		Ref		Ref	
9h<-10h	4.06 (1969)	1.02 (0.49-2.10)	0.967	0.75 (0.36-1.56)	0.441	0.71 (0.33-1.56)	0.401
>10h	16.32 (367.5)	4.02 (1.76-9.21)	0.001	2.70 (1.17-6.24)	0.020	2.65 (1.14-6.16)	0.023

Model 1: crude model.

Model 2: adjusted for age at baseline (<60 years, ≥60 years), residence (urban or rural) and education (illiteracy, primary school, middle school, high school or above).

Model 3 was further adjusted for BMI (<24kg/m2,≥24kg/m2), SBP (<140mmHg,≥140mmHg), smoking status (yes/no), alcohol consumption(yes/no), drinking tea(yes/no), drinking coffee(yes/no), total energy intake (continuous), total fat intake (continuous), total carbohydrate intake (continuous) and total protein intake (continuous).

BMI, body mass index; SBP, systolic blood pressure; HR, hazard ratio; CI, confidence interval.

### 3.3 Correlation between sleep duration and new-onset diabetes in male group

First, as shown in **Supplementary Figure 2**, we performed restricted cubic splines and found that, unlike women, there was no nonlinear relationship of sleep duration in men (p for non-linearity =0.7003). But we found a significant interaction (p for interaction=0.009) between sleep duration and age (<60 years, ≥60 years) (**Table 3**). This suggests that the association between sleep duration and new-onset diabetes may vary across different age groups in men. So, we conducted the study in two different age groups.

**Table 3:** Results of statistical interaction test between sleep duration and age.

Interactions	*P*
Age*sleep duration(Male)	0.009
Age*sleep duration (Female)	0.501

Age (<60 years, ≥60 years).*Sign of multiplication.

**Table 4** shows that the risk of diabetes decreased significantly with increasing sleep duration in men younger than 60 years, if sleep duration is considered as a continuous variable (per SD increment: HR, 0.75;95% CI, 0.60-0.94). This relationship was not found in older men over 60 years of age. In the way, the result showed that sleep duration had a negative effect on the incidence risk of new-onset diabetes when sleep duration was assessed as four groups. After controlling for residence, education level, BMI, SBP, smoking status, alcohol consumption, tea and coffee consumption and dietary intake (Energy, Carbohydrate, Fat, Protein), we found that the adjusted hazard ratios (HRs) and 95% confidence intervals (CIs) of diabetes in 7h-<8h/day, 8h-<9h/day, ≥9h/day were 0.73(0.42-1.24), 0.57(0.33-0.99) and 0.35(0.14-0.90), respectively, compared with participants who slept <7h/day (p-value for the trend <0.001) (**Table 5**). Further considering sleep duration as a categorical variable, no significant association between sleep duration and diabetes risk was observed in older men (age≥60)(**Supplementary Table 1**).

**Table 4:** Relationship between sleep duration (continuous variable) and new onset diabetes in men.

Sleep duration at groups	Model1		Model2		Model3	
	HR (95% CI)	P	HR (95% CI)	P	HR (95% CI)	P
age<60 years	0.67 (0.54-0.82)	<0.001	0.73 (0.59-0.92)	0.006	0.75 (0.60-0.94)	0.012
age≥60 years	1.02 (0.81-1.28)	0.898	1.15 (0.90-1.46)	0.257	1.17 (0.92-1.48)	0.210

Model 1: crude model.

Model 2: adjusted for residence (urban or rural) and education (Illiteracy, Primary school, middle school, high school or above), BMI (<24kg/m2,≥24kg/m2).

Model 3 was further adjusted for SBP (<140mmHg,≥140mmHg), smoking status (yes/no), alcohol consumption(yes/no), tea consumption (yes/no), coffee consumption(yes/no), total energy intake (continuous), total fat intake (continuous), total carbohydrate intake (continuous) and total protein intake (continuous).

BMI, body mass index; SBP, systolic blood pressure; HR, hazard ratio; CI, confidence interval.

**Table 5:** Relationship between sleep duration (categorical variable) and new-onset diabetes in men < 60 years old.

Sleep duration	Crude Model			Adjusted Model		
	HR (95% CI)	P	P for trend	HR (95% CI)	P	P for trend
<7h	Ref		1.27e-4	Ref		3.50e-2
7h-<8h	0.57 (0.34-0.95)	0.031		0.73 (0.42-1.24)	0.247	
8h-<9h	0.44 (0.26-0.73)	0.002		0.57 (0.33-0.99)	0.047	
≥9h	0.25 (0.11-0.60)	0.002		0.35 (0.14-0.90)	0.030	

Adjusted for residence (urban or rural) and education (illiteracy, primary school, middle school, high school or above), BMI (<24kg/m2,≥24kg/m2), SBP (<140mmHg,≥140mmHg), smoking status (yes/no), alcohol consumption(yes/no), drinking tea(yes/no), drinking coffee(yes/no), total energy intake (continuous), total fat intake (continuous), total carbohydrate intake (continuous) and total protein intake (continuous).

BMI, body mass index; SBP, systolic blood pressure; HR, hazard ratio; CI, confidence interval.

**Figure 2:**
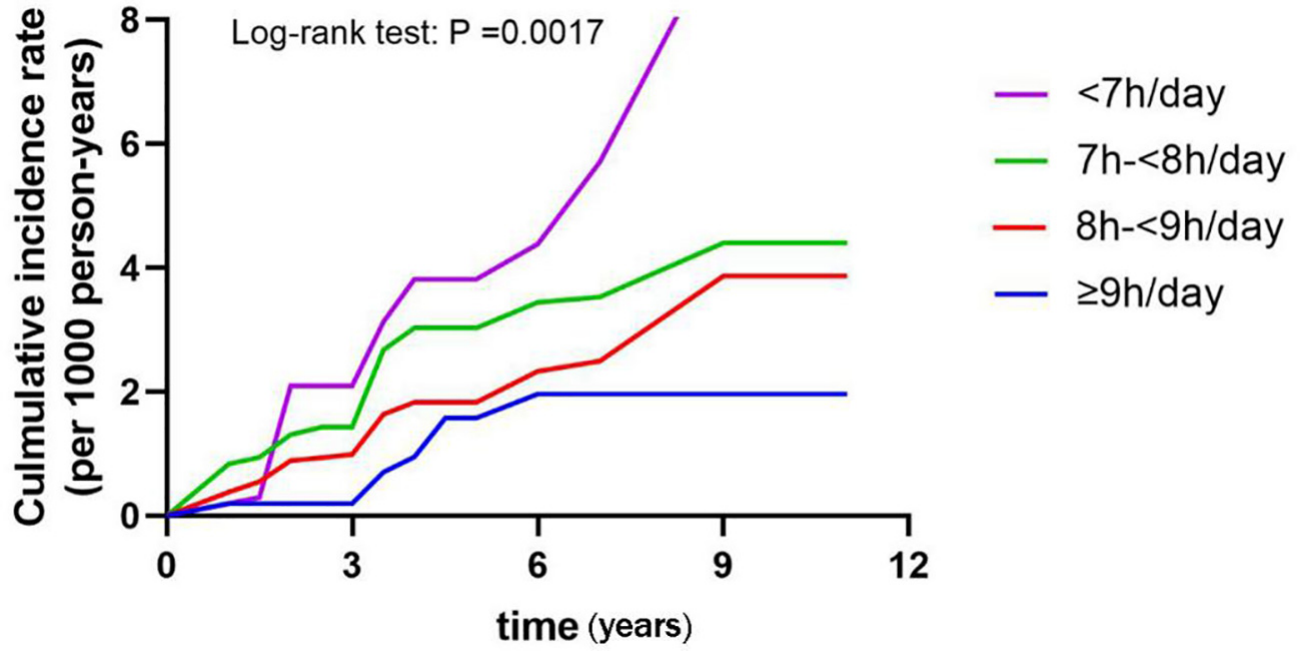
Cumulative incidence rates by sleep duration in different groups of men <60 years old.

Then, Kaplan-Meier analysis was used to explore the effect of sleep duration on the cumulative probability of diabetes (**Figure 2**). It was observed that those slept<7h/day had the highest cumulative incidence of diabetes (log-rank test: *P* =0.0017).

### 3.4 Sensitivity analyses

Sensitivity analyses were performed to test the robustness of the results. The same results were obtained when the missing values of BMI, SBP, smoking status, alcohol consumption, tea consumption, coffee consumption, MET and socioeconomic status were imputed with the use of multiple imputation (**Supplementary Tables 2**, **3**).

## 4 Discussion

This is a large retrospective cohort study that examined sex differences in the association between sleep duration and the risk of new-onset diabetes. Our findings are consistent with emerging evidence that sleep duration is closely associated with new-onset diabetes. But the effect was different between men and women. What’s more, our study emphasizes the necessity of considering gender disparities when examining this subject, by segregating males and females. It has been observed that women exhibit optimal sleep duration, while the results were different for older and non-older men.

The relationship between sleep duration and new-onset diabetes in women was U-shaped, and the risk of new-onset diabetes in women with sleep duration of 8h to 9h per day was the lowest. The risk of new-onset diabetes significantly decreased with the increment of sleep duration in participants with sleep duration ≤ 8h/day. Among women with sleep duration ≥9h/day, the risk of new-onset diabetes significantly increased with the increment of sleep duration. However, the relationship between sleep duration and the risk of diabetes was different in men and related to age. In adult men under 60 years of age, sleep duration was inversely and independently related to incident diabetes after adjusting for potential confounding variables. As sleep duration increased, the risk of new-onset diabetes tended to decrease. Moreover, it is worth noting that in older men the association between sleep duration and risk of new-onset diabetes appeared was not significant. This suggests that non-elderly men seem to be more concerned about their daily sleep duration. For men over 60, the focus should be directed towards established factors such as obesity, dietary patterns, waist circumference, and sedentary behavior rather than sleep duration. Whether the effect of sleep duration on diabetes is influenced by age may be an important point of difference between men and women. As observed in the **Table 3**, women had no significant interaction between sleep duration and age (< 60 years old and ≥ 60 years old).

Our findings are consistent with some previous studies. A multicenter cross-sectional study reported that both short and long sleep duration were associated with an increased risk of diabetes in middle-aged females (12). In addition, this U-shaped relationship between diabetes and sleep duration was also found in postmenopausal women in Philippines (18). Compared to women who slept 8-9h/day, women who slept less than 6h/day and more than 10h/day had a 2.47, 2.65 times higher risk of developing diabetes, respectively in this study. It appears that women who sleep longer (10h/day) have a higher risk of new-onset diabetes than those who sleep shorter (<6h/day). The effect of short sleep duration on diabetes is direct: insufficient sleep may induce insulin resistance and reduce insulin sensitivity (19); Short sleep duration increases sympathetic nerve activity, which inhibits insulin secretion from pancreatic cells, resulting in decreased glucose tolerance (20); Reduced sleep duration may be a new regulator of insulin signaling pathways (21); Lack of sleep can increase energy intake and lead to weight gain, which can affect blood sugar regulation (22). The contribution of longer sleep duration to diabetes risk was found only in women. The mechanisms by which excessive sleep duration leads to an increased risk of diabetes are not fully understood. Previous studies showed that women sleep longer but sleep is slightly less efficient (23). Moreover, women experience more insomnia problems and use more sleep medication than men (14). The potential impact of long sleep duration on the development of diabetes in women may be mediated indirectly.

In non-elderly men, the effect of sleep duration on diabetes risk needs to be interpreted with more caution. In some prior studies, a prominent association between short or long sleep duration and diabetes risk was confined to young individuals (24, 25). We also found it in the male population. For men under 60 years of age, the risk of diabetes may be reduced by 25% for every additional hour of sleep. Extending sleep indefinitely is certainly not advisable, but our findings encourage men under 60 to extend their sleep appropriately, as it appears that sleep deprivation, rather than long sleep duration, is associated with an increased risk of diabetes. Cedernae et al. (26) found that in male subjects, peripheral insulin resistance was significantly increased in those who were partially sleep deprived compared with those who slept through the night. In addition, studies of sleep restriction in men have shown that sleep disturbances lead to reduced circulating testosterone levels, which is harmful for glucose metabolism in men with diabetes (27, 28). Our study showed that the cumulative incidence of diabetes was highest in non-elderly men with sleep duration less than 7h/day. Short sleep duration in young healthy men is associated with reduced leptin levels, increased ghrelin levels, and increased hunger and appetite (29). Another studies have shown that extended sleep duration was associated with an improvement in insulin resistance and a reduction in the prevalence of abnormal fasting glucose (30, 31). This suggests potential benefit of sleep extension in preventing diabetes, which may explain the same trend observed in men under 60 years of age in our study.

The strengths of this study include the large population-based investigation and follow-up of sex differences in the associations between sleep duration and incidence of diabetes. To our knowledge, few such study has yet explored this relationship in Chinese people. However, there were also some limitations. Although we adjusted for some variables commonly associated with diabetes, we had to be limited by the selection of confounding variables in the database, and factors such as sleep quality, nap and shift work were not included in the study. In the present study, sleep duration and new onset events of diabetes were self-reported by the participants. This is a more subjective assessment of sleep duration than measured using objective methods, which may be subject to reporting and measurement bias. Our study was based on a Chinese population and may not be applicable to populations in other countries, suggesting that future studies should explore these associations in other ethnic and geographic groups.

## 5 Conclusion

In conclusion, sleeping too long or too short increases the risk of diabetes in women. The optimal sleep duration was 8-9h/day. Insufficient sleep, but not prolonged sleep, is a significant risk factor for developing diabetes among men under 60 years of age. Further studies with larger scale are needed to explore the mechanisms underlying the different associations between sleep duration and glucose metabolism in different genders and ages.

## Data Availability

The original contributions presented in the study are included in the article/**Supplementary Material**. Further inquiries can be directed to the corresponding authors.

## References

[1] Stanaway JD Afshin A Gakidou E Lim SS Abate D Abate KH. Global, regional, and national comparative risk assessment of 84 behavioural, environmental and occupational, and metabolic risks or clusters of risks for 195 countries and territories, 1990-2017: a systematic analysis for the Global Burden of Disease Study 2017. Lancet. (2018) 392(10159):1923–94. doi: 10.1016/S0140-6736(18)32225-6 PMC6227755 30496105

[2] Zheng Y Ley SH Hu FB. Global aetiology and epidemiology of type 2 diabetes mellitus and its complications. Nat Rev Endocrinol. (2018) 14:88–98. doi: 10.1038/nrendo.2017.151 29219149

[3] Li Y Teng D Shi X Qin G Qin Y Quan H. Prevalence of diabetes recorded in mainland China using 2018 diagnostic criteria from the American Diabetes Association: national cross-sectional study. BMJ. (2020) 369:m997. doi: 10.1136/bmj.m997 32345662 PMC7186854

[4] Wang L Peng W Zhao Z Zhang M Shi Z Song Z. Prevalence and treatment of diabetes in China, 2013-2018. JAMA. (2021) 326:2498–506. doi: 10.1001/jama.2021.22208 PMC8715349 34962526

[5] Shan Z Ma H Xie M Yan P Guo Y Bao W. Sleep duration and risk of type 2 diabetes: a meta-analysis of prospective studies. Diabetes Care. (2015) 38:529–37. doi: 10.2337/dc14-2073 25715415

[6] Yadav D Cho KH. Total sleep duration and risk of type 2 diabetes: evidence-based on clinical and epidemiological studies. Curr Drug Metab. (2018) 19:979–85. doi: 10.2174/1389200219666180628170431 29956620

[7] Jin X Chen Y Feng H Zhou M Chan JWY Liu Y. Association of accelerometer-measured sleep duration and different intensities of physical activity with incident type 2 diabetes in a population-based cohort study. J Sport Health Sci. (2024) 13(2):222ߝ32. doi: 10.1016/j.jshs.2023.03.001 PMC10980868 36871624

[8] Lu H Yang Q Tian F Lyu Y He H Xin X. A meta-analysis of a cohort study on the association between sleep duration and type 2 diabetes mellitus. J Diabetes Res. (2021) 2021:8861038. doi: 10.1155/2021/8861038 33834077 PMC8012145

[9] Maskarinec G Jacobs S Amshoff Y Setiawan VW Shvetsov YB Franke AA. Sleep duration and incidence of type 2 diabetes: the Multiethnic Cohort. Sleep Health. (2018) 4:27–32. doi: 10.1016/j.sleh.2017.08.008 29332675 PMC5771414

[10] Marks PA Monroe LJ. Correlates of adolescent poor sleepers. J Abnorm Psychol. (1976) 85:243–6. doi: 10.1037/0021-843X.85.2.243 1254786

[11] Oginska H Pokorski J. Fatigue and mood correlates of sleep length in three age-social groups: School children, students, and employees. Chronobiol Int. (2006) 23(6):1317–28. doi: 10.1080/07420520601089349 17190716

[12] Tuomilehto H Peltonen M Partinen M Seppä J Saaristo T Korpi-Hyövälti E. Sleep duration is associated with an increased risk for the prevalence of type 2 diabetes in middle-aged women - The FIN-D2D survey. Sleep Med. (2008) 9:221–7. doi: 10.1016/j.sleep.2007.04.015 17644479

[13] Cui S Li Y Chen Y Ren P Fan M Yang X. Association of sleep duration with risk of type 2 diabetes mellitus in a rural Chinese population: a nested case-control study. Sleep Breath. (2022) 26:2025–33. doi: 10.1007/s11325-021-02535-5 34839464

[14] Kocevska D Lysen TS Dotinga A Koopman-Verhoeff ME Luijk MPCM Antypa N. Sleep characteristics across the lifespan in 1.1 million people from the Netherlands, United Kingdom and United States: a systematic review and meta-analysis. Nat Hum Behav. (2021) 5:113–22. doi: 10.1038/s41562-020-00965-x 33199855

[15] Wang J Kwok MK Au Yeung SL Li AM Lam HS Leung JYY. Sleep duration and risk of diabetes: Observational and Mendelian randomization studies. Prev Med. (2019) 119:24–30. doi: 10.1016/j.ypmed.2018.11.019 30508554

[16] Li W Jiao Y Wang L Wang S Hao L Wang Z. Association of serum magnesium with insulin resistance and type 2 diabetes among adults in China. Nutrients. (2022) 14(9):1799. doi: 10.3390/nu14091799 35565766 PMC9104014

[17] Kivimäki M Virtanen M Kawachi I Nyberg ST Alfredsson L Batty GD. Long working hours, socioeconomic status, and the risk of incident type 2 diabetes: a meta-analysis of published and unpublished data from 222 120 individuals. Lancet Diabetes Endocrinol. (2015) 3(1):27–34. doi: 10.1016/S2213-8587(14)70178-0 25262544 PMC4286814

[18] Ayas NT White DP Al-Delaimy WK Manson JE Stampfer MJ Speizer FE. A prospective study of self-reported sleep duration and incident diabetes in women. Diabetes Care. (2003) 26:380–4. doi: 10.2337/diacare.26.2.380 12547866

[19] Broussard JL Chapotot F Abraham V Day A Delebecque F Whitmore HR. Sleep restriction increases free fatty acids in healthy men. Diabetologia. (2015) 58:791–8. doi: 10.1007/s00125-015-3500-4 PMC4358810 25702040

[20] Rao MN Neylan TC Grunfeld C Mulligan K Schambelan M Schwarz JM. Subchronic sleep restriction causes tissue-specific insulin resistance. J Clin Endocrinol Metab. (2015) 100:1664–71. doi: 10.1210/jc.2014-3911 PMC4399283 25658017

[21] Broussard JL Ehrmann DA Van Cauter E Tasali E Brady MJ. Impaired insulin signaling in human adipocytes after experimental sleep restriction: a randomized, crossover study. Ann Intern Med. (2012) 157:549–57. doi: 10.7326/0003-4819-157-8-201210160-00005 PMC4435718 23070488

[22] Hibi M Kubota C Mizuno T Aritake S Mitsui Y Katashima M. Effect of shortened sleep on energy expenditure, core body temperature, and appetite: a human randomised crossover trial. Sci Rep. (2017) 7:39640. doi: 10.1038/srep39640 28071649 PMC5223114

[23] Kerkhof GA. Epidemiology of sleep and sleep disorders in The Netherlands. Sleep Med. (2017) 30:229–39. doi: 10.1016/j.sleep.2016.09.015 28215254

[24] Heianza Y Kato K Fujihara K Tanaka S Kodama S Hanyu O. Role of sleep duration as a risk factor for Type 2 diabetes among adults of different ages in Japan: the Niigata Wellness Study. Diabetes Med. (2014) 31:1363–7. doi: 10.1111/dme.2014.31.issue-11 25124930

[25] Song Q Liu X Zhou W Wang X Wu S. Short-term changes in sleep duration and risk of type 2 diabetes: Kailuan prospective study. Med (Baltimore). (2016) 95:e5363. doi: 10.1097/MD.0000000000005363 PMC5106068 27828862

[26] Cedernaes J Lampola L Axelsson EK Liethof L Hassanzadeh S Yeganeh A. A single night of partial sleep loss impairs fasting insulin sensitivity but does not affect cephalic phase insulin release in young men. J Sleep Res. (2016) 25:5–10. doi: 10.1111/jsr.2016.25.issue-1 26361380

[27] Reynolds AC Dorrian J Liu PY Van Dongen HP Wittert GA Harmer LJ. Impact of five nights of sleep restriction on glucose metabolism, leptin and testosterone in young adult men. PLoS One. (2012) 7:e41218. doi: 10.1371/journal.pone.0041218 22844441 PMC3402517

[28] Cai X Tian Y Wu T Cao CX Li H Wang KJ. Metabolic effects of testosterone replacement therapy on hypogonadal men with type 2 diabetes mellitus: a systematic review and meta-analysis of randomized controlled trials. Asian J Androl. (2014) 16:146–52. doi: 10.4103/1008-682X.122346 PMC3901874 24369149

[29] Spiegel K Tasali E Penev P Van Cauter E. Brief communication: Sleep curtailment in healthy young men is associated with decreased leptin levels, elevated ghrelin levels, and increased hunger and appetite. Ann Intern Med. (2004) 141:846–50. doi: 10.7326/0003-4819-141-11-200412070-00008 15583226

[30] Leproult R Deliens G Gilson M Peigneux P. Beneficial impact of sleep extension on fasting insulin sensitivity in adults with habitual sleep restriction. Sleep. (2015) 38:707–15. doi: 10.5665/sleep.4660 PMC4402666 25348128

[31] So-Ngern A Chirakalwasan N Saetung S Chanprasertyothin S Thakkinstian A Reutrakul S. Effects of two-week sleep extension on glucose metabolism in chronically sleep-deprived individuals. J Clin Sleep Med. (2019) 15:711–8. doi: 10.5664/jcsm.7758 PMC6510689 31053213

