# Supplementary material for: Gender differences in the association between sleep duration and diabetes in Chinese adults
Source: Front Endocrinol (Lausanne). 2025 May 20;16:1385618. doi: 10.3389/fendo.2025.1385618 (PMC12129752; doi:10.3389/fendo.2025.1385618)
Supplement: Supplementary file 1 [file DataSheet1.docx]

Supplementary figure 1: Flow chart


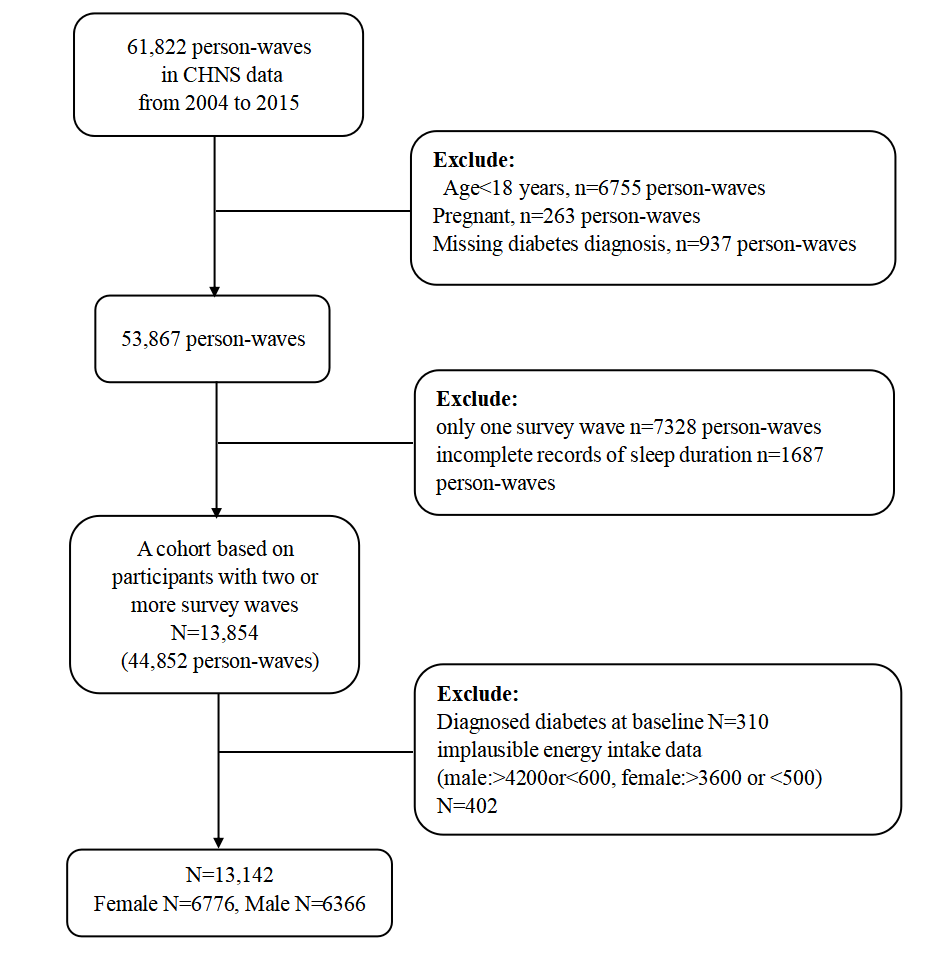


Supplementary figure 2: Restricted cubic splines of sleep hours associated with risk of new-onset diabetes in men（p for non-linearity =0.7003)


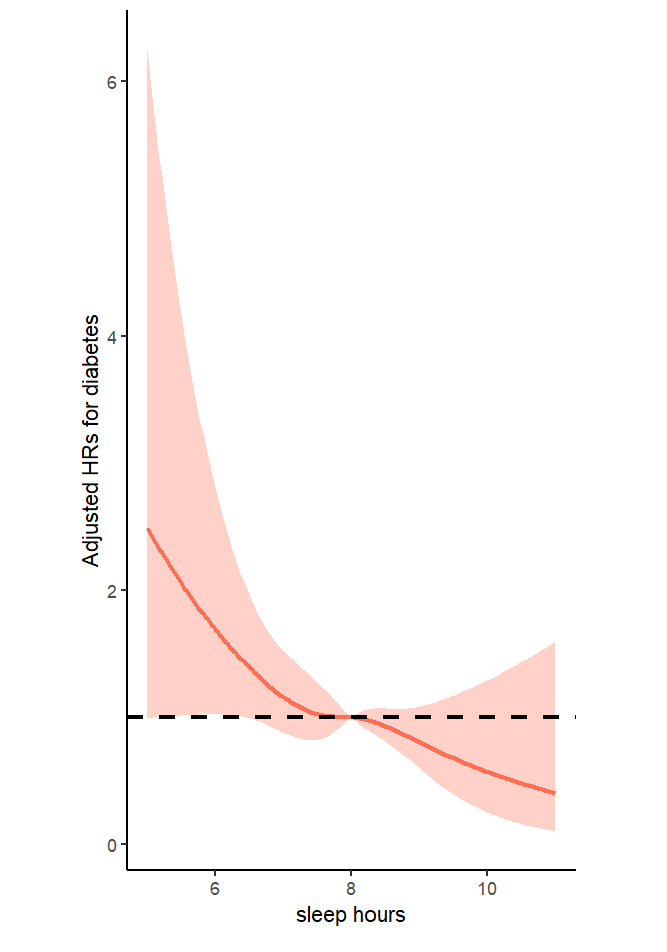


Adjusted for age at baseline (<60 years, ≥60 years), residence (urban or rural) and education (illiteracy, primary school, middle school, high school or above), BMI (<24kg/m2,≥24kg/m2), SBP (<140mmHg,≥140mmHg), smoking status (yes/no), alcohol consumption(yes/no), drinking tea(yes/no), drinking coffee(yes/no), total energy intake (continuous), total fat intake (continuous), total carbohydrate intake (continuous) and total protein intake (continuous).
