# Supplementary material for: Gender differences in the association between sleep duration and diabetes in Chinese adults
Source: Front Endocrinol (Lausanne). 2025 May 20;16:1385618. doi: 10.3389/fendo.2025.1385618 (PMC12129752; doi:10.3389/fendo.2025.1385618)
Supplement: Supplementary file 2 [file Table1.docx]

Supplementary Table1

Relationship between sleep duration (categorical variable) and new-onset diabetes in men ≥60 years old

| Sleep duration | Crude Model | | | Adjusted Model | | |
| --- | --- | --- | --- | --- | --- | --- |
|  | HR (95% CI) | P | P for trend | HR (95% CI) | P | P for trend |
| <7h | Ref |  | 0.796 | Ref |  | 0.488 |
| 7h-<8h | 1.16(0.47-2.86) | 0.746 |  | 1.33(0.50-3.55) | 0.575 |  |
| 8h-<9h | 1.34(0.56-3.21) | 0.513 |  | 1.93(0.74-5.03) | 0.179 |  |
| ≥9h | 0.85(0.30-2.40) | 0.763 |  | 1.33(0.43-4.05) | 0.619 |  |

Adjusted for residence (urban or rural) and education (Illiteracy, Primary school, middle school, high school or above), BMI (<24kg/m2,≥24kg/m2), SBP (<140mmHg,≥140mmHg), DBP (<90mmHg,≥90mmHg), smoking status (yes/no), alcohol consumption(yes/no), drinking tea(yes/no), drinking coffee(yes/no), total energy intake (continuous), total fat intake (continuous), total carbohydrate intake (continuous) and total protein intake (continuous).

Abbreviations: BMI, body mass index; SBP, systolic blood pressure; DBP, diastolic blood pressure; HR, hazard ratio; CI, confidence interval

Supplementary Table 2

Relationship between sleep duration (categorical variable) and new-onset diabetes in women

| Sleep duration | Model1 | | Model2 | | Model3 | |
| --- | --- | --- | --- | --- | --- | --- |
|  | HR (95% CI) | P | HR (95% CI) | P | HR (95% CI) | P |
| <6h | 4.71（2.36, 9.30） | ＜0.001 | 2.77(1.39,5.58) | ＜0.001 | 2.59(1.28,5.21) | 0.008 |
| 6h-<7h | 3.13(2.12, 4.71) | ＜0.001 | 2.18(1.45,3.32) | 0.004 | 1.77(1.15,2.69) | 0.008 |
| 7h-<8h | 1.48(1.12,1.97) | 0.010 | 1.36(1.12,1.82) | 0.042 | 1.35(0.99,1.82) | 0.055 |
| 8h-9h | Ref |  | Ref |  | Ref |  |
| 9h<-10h | 1.02(0.49,2.10) | 0.967 | 0.75(1.36,1.55) | 0.441 | 0.74(0.34,1.61) | 0.449 |
| >10h | 4.06(1.77,9.21) | 0.001 | 2.69(1.19,6.23) | 0.020 | 1.04(0.20,1.88) | 0.016 |

Missing values for BMI, SBP, DBP, smoking status, alcohol consumption, drinking tea, drinking coffee, MET, socioeconomic status were imputed with the use of multiple imputation.

Model 2: adjusted for age at baseline (<60, ≥60), residence (urban or rural) and education (Illiteracy, Primary school, middle school, high school or above).

Model 3 was further adjusted for BMI (<24kg/m2,≥24kg/m2), SBP (<140mmHg,≥140mmHg), DBP (<90mmHg,≥90mmHg), smoking status (yes/no), alcohol consumption(yes/no), drinking tea(yes/no), drinking coffee(yes/no), total energy intake (continuous), total fat intake (continuous), total carbohydrate intake (continuous), total protein intake (continuous), MET(Low, medium, high), socioeconomic status (low, medium, high).

Abbreviations: BMI, body mass index; SBP, systolic blood pressure; MET, metabolic in counts of tasks; HR, hazard ratio; CI, confidence interval

Supplementary Table 3

Relationship between sleep duration (categorical variable) and new-onset diabetes in men < 60 years old

| Sleep duration | Crude Model | | | Adjusted Model | | |
| --- | --- | --- | --- | --- | --- | --- |
|  | HR (95% CI) | P | P for trend | HR (95% CI) | P | P for trend |
| <7h | Ref |  | 7.47e-6 | Ref |  | 4.30e-6 |
| 7h-<8h | 0.57(0.35,0.61) | 0.031 |  | 0.67(0.39,1.15) | 0.150 |  |
| 8h-<9h | 0.44(0.26,0.73) | 0.002 |  | 0.52(0.31,0.92) | 0.025 |  |
| ≥9h | 0.25(0.29,0.59) | 0.002 |  | 0.33(0.13,0.85) | 0.021 |  |

Missing values for BMI, SBP, smoking status, alcohol consumption, drinking tea, drinking coffee, MET, socioeconomic status were imputed with the use of multiple imputation.

Adjusted for residence (urban or rural) and education (Illiteracy, Primary school, middle school, high school or above), BMI (<24kg/m2,≥24kg/m2), SBP (<140mmHg,≥140mmHg), DBP (<90mmHg,≥90mmHg), smoking status (yes/no), alcohol consumption(yes/no), drinking tea(yes/no), drinking coffee(yes/no), total energy intake (continuous), total fat intake (continuous), total carbohydrate intake (continuous), total protein intake (continuous), MET(Low, medium, high), socioeconomic status (low, medium, high).

Abbreviations: BMI, body mass index; SBP, systolic blood pressure; MET, metabolic in counts of tasks; HR, hazard ratio; CI, confidence interval
